# Efficient CO_2_ methanation using nickel nanoparticles supported mesoporous carbon nitride catalysts

**DOI:** 10.1038/s41598-023-31958-1

**Published:** 2023-03-24

**Authors:** Zakaria Refaat, Mohamed El Saied, Ahmed O. Abo El Naga, Seham A. Shaban, H B Hassan, Mohamed Refaat Shehata, F. Y. El Kady

**Affiliations:** 1 Catalysis Department, Refining Division, Egyptian Petroleum Research Institute, Nasr City, 11727 Cairo Egypt; 2 Chemistry Department, Faculty of Science, Cairo University, Giza, Egypt

**Keywords:** Chemistry, Energy science and technology

## Abstract

The CO_2_ methanation technique not only gives a solution for mitigating CO_2_ emissions but can also be used to store and convey low-grade energy. The basic character and large surface area of mesoporous carbon nitride, (MCN), are considered promising properties for the methanation of CO_2_. So, a series (5–20 wt.%) of Ni-doped mesoporous carbon nitride catalysts were synthesized by using the impregnation method for CO_2_ methanation. the prepared catalysts were characterized by several physicochemical techniques including XRD, BET, FT-IR, Raman spectroscopy, TEM, TGA analysis, Atomic Absorption, H_2_-TPR, and CO_2_-TPD. The catalytic performance was investigated at ambient pressure and temperature range (200–500 °C) using online Gas chromatography system. The prepared catalysts showed good performance where 15%Ni/MCN exhibited the best catalytic conversion and methane yield with 100% methane selectivity at 450 °C for investigated reaction conditions.

## Introduction

Global CO_2_ emissions have noticeably increased in recent years due to anthropic activity, which is considered one of the main reasons for global warming. Therefore, the world needs to reduce carbon emissions to stop the terrible increase in the concentration of carbon dioxide in the atmosphere^1^. As a result, one of the main challenges to mitigating global warming is creating economic opportunities through the production of renewable, safe, economic, and reliable clean energy^2^. CO_2_ utilization and conversion into valuable compounds are considered one of the most important of these techniques to overcome the global warming challenge. So, the conversion of CO_2_ to methane, methanol, or higher alcohols has gained great attention because of its usage in the so-called power-to-gas technology^3,4^. This methanation process, called the Sabatier reaction, is a catalytic reaction between CO_2_ and H_2_ gas in the presence of a metal-doped heterogeneous catalyst at pressures up to 100 bars and a temperature range between 150 °C and 600 °C, and heating above this limit should be avoided to prevent the catalyst deactivation^5^. This reaction depends on the reduction of CO_2_ by hydrogen gas which can be obtained from a renewable source (e.g. water electrolysis and biomass) or a nonrenewable source^6,7^. Methane, the major product of this process, is used extensively in the industry and in our daily life. It is considered an ample and clean source for the production of both shale and natural gaseous^8^. Thanks to its highest power value (about 56 kJ g^−1^) and the best combustion heat as compared to the other hydrocarbons, it has favorable potential in several applications^8,9^. Generally, it is a feasible fuel in the transportation sector, in the industry as an environmentally friendly raw material for chemical and petrochemical processes, and in the residential sector as a synthetic natural gas^10,11^. Furthermore, the production of methanol or liquid hydrocarbons from syngas via the Fischer–Tropsch process is considered the most popular methane conversion process^12^. According to all of these methane benefits, the CO_2_ methanation process has significant importance not only for CO_2_ concentration reduction in the atmosphere but also for the production of a meaningful and valuable product.

From a thermodynamic point of view, this reaction is exothermic, releases a large amount of heat, and can be expressed as follows^13^

1 $${\text{CO}}_{{2}} + {\text{ 4H}}_{{2}} \to {\text{CH}}_{{4}} + {\text{ 2H}}_{{2}} {\text{O}} \quad \Delta {\text{H }} = \, - {\text{164 kJmol}}^{{ - {1}}}$$

From the standpoint of dynamics, this reduction is an eight-electron reaction, which leads to the obvious kinetic barrier. As a result, it needs high energy to overcome this barrier and decrease the stable CO_2_ with a strong C = O bond into CH_4_ (− 4)^14,15^. Since the composition of the catalyst has a significant influence on the reaction's performance, the doped catalysts with a single metal or more exhibit high activity^16^. Various transition and noble metals doped on metal oxides and mesoporous materials can be used for the CO_2_ methanation reaction. According to previous reports, the majority of metals in the VIII group could serve as active sites for CO_2_ methanation. Most of these metals—Ru, Pd, and Pt—are highly precious metals. The high cost and low availability of these noble metals are considered the main drawbacks that inhibit their use on an industrial scale. So, the transition metals such as Ni, Fe, and Co with relatively low reactivity were used for this purpose^17,18^. High Ni-doped heterogeneous catalysts are also known to have high intrinsic activity, high CH_4_ selectivity, high availability, and low cost^19^ but metal particle sintering and carbon formation on the catalyst surface at high temperatures are regarded as their main drawbacks^20,21^. So, several supports, such as Al_2_O_3_, the commonly used support with nickel^22^, SiO_2_^23,24^, TiO_2_^25^, and porous materials such as metal–organic frameworks (MOFs)^26,27^, zeolites^28,29^, carbon nanotubes^30,31^, carbon nanofibers^32,33^, MCM-41^34^ and SBA-15^11^ are used with nickel for this reaction.

Due to their perfect structure, mesoporous materials are considered the best novel structures for methanation reactions, not only due to their high specific surface area but also due to their ability to prevent the sintering of active sites on their surfaces, as reported by Shen et al. In recent years, two-dimensional graphitic mesoporous carbon nitride (gmp-C_3_N_4_) has been investigated in broad catalytic fields due to its tunable electronic character, semi conductivity, and a unique structure that is similar to the graphene structure, in addition to the Lewis base properties as a result of nitrogen richness, which provide good nickel dispersion^35,36^. It appears to have good performance as a support material for heterogeneous catalysts in a lot of thermochemical reactions. It has been used as a metal-free catalyst in several reactions, such as oxidation^37^ and photocatalytic reactions^38^, and as a supporting material doped with metals for electrocatalytic^39^ and hydrogenation reactions^40^. It consists of polymer chains of s-triazine or tri-s-triazine (heptazine) aromatic rings, which comprise primarily covalently, bonded carbon and nitrogen. These strong covalent bonds enable it to be highly stable in both acidic and basic mediums^41,42^. Furthermore, the aromatic rings in the structure result in high thermal and electronic structures^43^. In the case of hydrogenation applications, MCN has several favorable properties that enable it to be a suitable support for these applications. Particularly for CO_2_ methanation, the hydrophilicity, and the high basicity have a significant effect on the nickel nanoparticles dispersion. Furthermore, the Improvability of morphology could enhance the catalytic performance^36,44^.

In addition, supports of mixed metal oxides are used for this reaction to improve the reactivity of the catalyst^45,46^. The addition of promoters is considered an additional effort used to improve the catalytic activity. The oxides of alkaline-earth metals are also the more abundant oxides used for this purpose, especially the oxides of Ca, Ba, and Mg^47,48^. For nickel-based catalysts, the strong interaction between Ni and support, the perfect metal dispersion on the support surface, and catalyst morphology are important factors that affect the conversion efficiency of the catalyst^49,50^. The good dispersion of nickel on the support is usually achieved by using micro or mesoporous supports that have good structural properties and have the ability for modifier addition^51^. The formation of CH_4_ on the surface of the catalyst is controlled by several factors, but the electron density of the active sites is considered the most important factor. The electron transfer between the active sites and the support will increase the electron density and enhance the nickel–carbon coupling, which leads to the breaking of bonds in the carbonyl group and the formation of methane^52^.

Several porous materials have been subjected to extensive research for this purpose continuously, owing to the importance of the structural properties of the catalyst for CO_2_ methanation. Besides, carbonaceous materials such as activated carbon, carbon nanotubes, graphene, and carbon nitrides have attracted considerable interest in CO_2_ capture and methanation due to their superior chemical and electrical properties, efficient adsorption capacity, thermal stability, and exemplary morphology. In this regard, Mesoporous carbon nitride is an intriguing material with exceptional chemical, thermal, and mechanical capabilities. Not only does it possess a large specific surface area, mesoporosity with pore diameters ranging from 2 to 50 nm, nitrogen group richness, and semiconductivity with a bandgap energy of 2.5–2.8 eV, but it also has additional attractive properties such as easy surface improvement by incorporation of metal nanoparticles such as nickel, iron, and cubber; its structure is easily modified by protonation or alkylation due to the N–H bonds in its structure, and because of the abundance of nitrogen atoms, it can serve as a Lewis base due to nitrogen lone pairs of electrons. Additionally, the SP^2^ hybridization of carbon and nitrogen atoms culminates in a layered structure of π-conjugated graphitic planes^37^.

Because of its unique features for a range of applications in lucrative domains such as electro-^53^, basic-, and photocatalysis^53^, supercapacitor^54^, energy storage^55^, conversion^56^, and sensing^57^, MCN has long attracted the curiosity of specialists. Following that, even though MCN has been extensively investigated and demonstrated to have a perfect performance in the field of photocatalytic CO_2_ conversion due to its unique properties in addition to its semiconductivity, mesoporous carbon nitride (gmp-C_3_N_4_) has not been extensively studied in the thermal hydrogenation of CO_2_^58^. In contrast to the other investigated applications for MCN, the utilization of Ni-doped mesoporous carbon nitride in thermal CO_2_ methanation is regarded as relatively recent. Substantial studies on CO_2_ thermal methanation employing monometallic nickel-doped mesoporous carbon nitride revealed low performance, needing more inquiry and development to reach the greatest CO_2_ conversion Percentage as well as flawless CH_4_ selectivity. Izabela S. Pieta et al. study's exemplifies ongoing research into the performance of Ni-doped carbon nitride in CO_2_ methanation^58^. According to Izabela S. Pieta et al., under atmospheric pressure, CO_2_ conversion and selectivity for light hydrocarbons, such as methane, increase as the temperature rises. The Ni/CN catalyst displayed CO_2_ conversion and methane selectivity of 20% and 80%, respectively, at 623 K, while performance improved to 44% at 750 K while maintaining Methane selectivity. Furthermore, Khairul et al.^59^ evaluated the efficacy of monometallic nickel supported on exfoliated graphitic carbon nitride for highly selective CO and CO_2_ methanation, as well as the effect of incorporating La or Ce with the nickel nanoparticles, in the same context. In the instance of CO_2_ methanation, the nickel catalyst performs best at 430 °C, with around 44% conversion and 95% methane selectivity.

Bearing in mind all of these promising attempts to identify carbon nitride compounds for this thermal application, the purpose of this work was to evaluate the use of carbon nitride with a mesoporous structure (gmp-C_3_N_4_) as a support for nickel nanoparticles to obtain novel catalysts for improving the thermal methanation of CO_2_. The produced catalysts will be characterized using a variety of analytical methods, including XRD, BET, TEM, IR, Raman, CO_2_-TPD, H_2_-TPR, Atomic Absorption, and TGA analyses. Subsequently, using an online gas chromatography system, The prepared catalysts will be evaluated as monometallic heterogeneous catalysts for CO_2_ methanation at temperatures ranging from 200 to 500 °C and with various weight ratios of nickel nanoparticles (5–20 wt%) to examine the CO_2_ conversion, methane selectivity and yield, and catalyst short-term stability.

## Experimental section

### Preparation of catalysts

#### Materials

Pluronic P123 copolymer (99%), hydrochloric acid (HCl, 36.5%), cetyltrimethylammonium bromide (CTAB, 99%), ethyl alcohol (C_2_H_5_OH, absolute), and ethylenediamine (EDA, ≥ 99%) were purchased from Sigma-Aldrich. Tetraethyl orthosilicate (TEOS, 98%), carbon tetrachloride (CTC, ≥ 99.5), and hydrofluoric acid (HF, 48%) were purchased from Merck. Nickel (II) chloride hexahydrate, (NiCl_2_.6H_2_O, 99.9%), Sodium borohydride (NaBH_4_, ≥ 98.0%), and nickel standard solution (1000 mg/L) for Atomic Absorption spectrometer were purchased from Scharlau. All of these chemicals were used without any further purification.

#### Silica hard template preparation

The silica hard template, SBA-15, was prepared as reported in the literature^60^. SBA-15 was prepared using Pluronic P123 copolymer, CTAB, and TEOS as a structure-directing agent, co-template, and silica precursor, respectively. Firstly, a homogenous solution of 16 ml purified water, 20 ml 2 M HCl, and 10 ml absolute ethanol was prepared. On stirring at 40 °C, 1.2 g of Pluronic P123 and 0.2 g of CTAB were dissolved into the homogenous solution. Under vigorous stirring at 40 °C, 4 mL of the silica precursor, TEOS, was added slowly and dropwise to the solution. The sample was left at this temperature for 45 min and then aged at 80 °C under reflux for 6 h to obtain self-assembly. The sample was then washed, filtered, and dried overnight at 70 °C and, after that, obeyed calcination in airflow at 540 °C for 5 h with ramping at 5 °C/min.

#### Mesoporous carbon nitride support preparation

In a typical synthesis, 0.5 g of SBA-15 was added to a mixture of ethylenediamine (EDA) and carbon tetrachloride (CTC) in the amounts of 1.35 g and 3 g, respectively. The product was then refluxed at 90 °C for 6 h. The obtained dark-brown solid was then dried in an oven at 60 °C overnight. The sample was then ground into a fine powder as a pre-calcination step. The calcination step is done in nitrogen flow with 5 °C /min ramping to 600 °C and then left at 600 °C for 5 h. To obtain pure mesoporous carbon nitride material, the sample was washed with HF (5%) to dissolve the remaining silica template, then filtered, washed with purified water and ethanol, and dried in an oven at 100 °C^61^.

#### Nickel nanoparticle preparation

In the typical process, 0.1 g of sodium borohydride (NaBH_4_) was added to 0.2 g of nickel chloride hexahydrate (NiCl_2_.6H_2_O) in a mortar and ground adequately until the ground powder became black. Then 10 ml of purified water was added slowly, and the hydrogen gas evolved immediately. After hydrogen release, the black precipitate was filtered, washed with purified water several times, and left to dry in the air naturally. The sample was then calcinated in hydrogen gas at 500 °C for 4 h^62^.

#### Catalysts preparation

In the typical process, the nickel nanoparticles are loaded onto the mesoporous carbon nitride support (MCN) by the addition of one gram of MCN into a beaker containing 50 ml of methanol, sonicated, and stirred for 45 min. The sample was then sonicated for 45 min and stirred for 45 min, three times, respectively. The sample was left on stirring at 60 °C for 4 h to evaporate the methanol and subsequently transferred to the oven for drying overnight, and eventually calcined in a nitrogen atmosphere at 600 °C for 2 h^59^.

### Catalysts characterization

X-ray diffraction (XRD) samples were identified by Bruker AXS D8-advanced diffractometer using Ni filtered Cu Kα radiation at 40 kV and 40 mA and the scan rate was 4˚per minute from 5° to 80°. For N_2_ adsorption–desorption isotherms, the samples were degassed firstly at 250 °C for 12 h and subsequently, at −196 °C = 77 K (liquid nitrogen temperature) the measurements were performed using a Quantachrome Nova 3200 S instrument. The Specific surface area (S_BET_) and pore size distributions were calculated by the BET and BJH method methods respectively.

The morphologies of the samples were characterized by a JEOL JEM-2100F instrument operated at 200 kV acceleration voltages for transmission electron microscopy (TEM) patterns.

FT-IR spectra of the samples were performed by (NEXUS, 670) FT-IR device after dilution of the samples with KBr powder and grinding, then pressing the samples into tablets. The measurements were taken by 16 scans within the range of 400–4000 cm^−1^ and 4 cm^−1^ resolution. Sentera Spectrometer was used for Raman spectroscopy analysis of the prepared samples in the range of 45–4500/cm.

SDT Q600, a thermal analysis module controlled by Q Series software, was used to perform thermal analysis on the TGA and DTG samples. In an alumina crucible, 100 mg of the sample is measured under a 100 ml/min flow of nitrogen atmosphere with a heating rate of 10 °C /min to 1000 °C. The catalyst's reducibility was assessed using H_2_-TPR analysis, which was carried out on a Micromeritics AutoChem II 2920 Plus Chemisorption analyzer equipped with a thermal conductivity detector (TCD). Nickel loadings were determined using Flame Atomic Absorption Spectrophotometer model Zenit 700p according to ASTM D4691.

To evaluate the basicity of the samples, CO_2_-TPD was assessed using the Belcat II instrument. 0.10 g of each sample was cleaned before examination by heating them at 500 °C for 2 h to remove any adsorbed impurities. Following cooling, the samples were saturated with CO_2_ and heated under helium flow at a rate of 5 °C/min. The quantity of desorbed CO_2_ gas was determined using a thermal conductivity detector (TCD).

### CO_2_ methanation performance assessment

The catalyst activity of the synthesized materials towards CO_2_ methanation was performed in a fixed bed reactor while the product was detected by an online GC system equipped with TCD (Agilent). Firstly, 100 mg of the sample was diluted with 1.0 g of the porcelain located in the reactor, and subsequently, we pretreated it in the reactor by passing the flow of H_2_/N_2_ mixture (25 ml min^−1^) at 450 °C for 2 h. After this activation process, the system was left to cool to 200 °C in the N_2_ atmosphere, and a mixture of H_2_ and CO_2_ gases by the molar percentage of H_2_/CO_2_ = 4/1 and a flow rate of 200 ml min^−1^was then fed into the reactor at the required temperature. The catalytic performance tests were evaluated at different temperatures (200 – 500 °C)^63^ and ambient pressure.

The CO_2_ conversion (X_CO2_), methane selectivity (S_CH4_), and methane yield (Y_CH4_) were calculated by the following equations^54^:

2 $$\left( {{\text{CO}}_{2} \,{\text{Conversion}}\;\left( {{\text{X}}_{{{\text{CO2}}}} } \right)\left( \% \right)} \right) = \frac{{{\text{n}}_{{{\text{CO2}},in}} - {\text{n}}_{{{\text{CO2}},{\text{out}}}} }}{{{\text{n}}_{{{\text{CO2,in}}}} }} \times 100$$

3 $$\left( {{\text{Methane}}\,{\text{selectivity}}\;\left( {{\text{S}}_{{{\text{CH4}}}} } \right)\left( \% \right)} \right) = \frac{{{\text{n}}_{{{\text{CH4,out}}}} }}{{{\text{n}}_{{{\text{CO2,in}}}} - {\text{n}}_{{{\text{CO2,out}}}} }} \times 100$$

4 $$\left( {{\text{Methane}}\,{\text{yield}}\;({\text{Y}}_{{{\text{CH4}}}} )(\% )} \right) = \frac{{{\text{n}}_{{{\text{CH4,out}}}} }}{{{\text{n}}_{{{\text{CO2,in}}}} }} \times 100$$

where, n_CO2, in_ and n_CO2, out_ are the molar flow rates (mol s^−1^) of CO_2_ at the reactor's inlet and outlet, respectively. Additionally, n_CH4, out_ is the molar flow rate (mol s^−1^) of CH_4_ at the reactor's outlet.

## Results and discussion

### Catalyst characterization

#### XRD analysis

**Figure 1:**
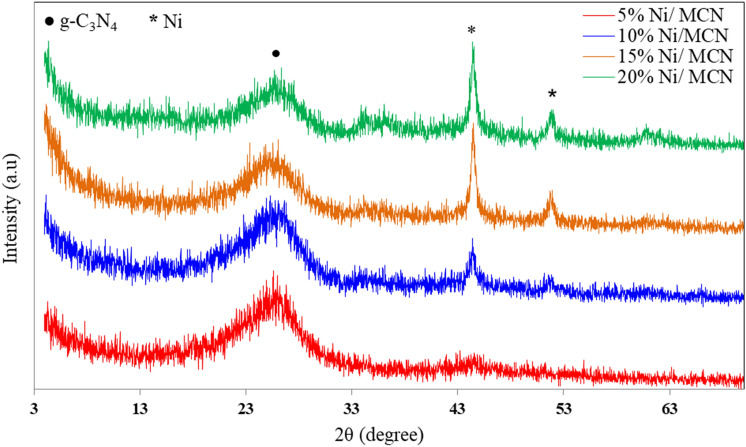
XRD charts of the prepared samples.

In this technique, the XRD patterns demonstrate the crystal structure of the nickel-doped MCN heterogeneous catalysts. The X-ray diffraction patterns of the prepared materials have been shown in Fig. 1. All of the samples revealed a hump peak at 25.9°, which is compatible with the g-C_3_N_4_ characteristic peak, which may be ascribed to the MCN (002) reflection plane^64^. This peak, in particular, is indicative of MCN's graphitic origin because it is a characteristic of graphitic materials^58^. As a consequence, because it is allocated from the planes generated by the agglutination of the connected aromatic groups in the layered structure, it is evidence of MCN and graphene's layered structural similarities^65,66^. Furthermore, as shown in Fig. 1, the nickel-doped catalysts show preservation of the (002) reflection plane at 2θ = 25.9° with a decrease in intensity by increasing the nickel nanoparticle concentration on the support surface, and this implies that the nickel nanoparticles' implantation does not influence the MCN's crystal structure since the spacing between the g-C_3_N_4_ layers stays unchanged^65^. Moreover, the patterns of the samples show the diffraction peaks of nickel nanoparticles at 44.5° and 51.8° related to the (111) and (200) reflection planes, respectively (PDF no. 01-087-0712, ICDD)^67,68^. Additionally, the intensities of these peaks were found to increase as the concentration of nickel nanoparticles increased. Furthermore, the absence of diffraction peaks of NiO at 37.3°, 43.3°, and 62.9° (PDF no. 030656920, ICCD) refers to the successful preparation of zero-valent nickel on the support surface. Please note that the lone pairs of electrons at the nitrogen atoms in the support structure act as electron donors and enhance the formation of zero-valent nickel nanoparticles that are dispersed onto the surface of the support^69^.

#### N_2_-physisorption analysis

**Figure 2:**
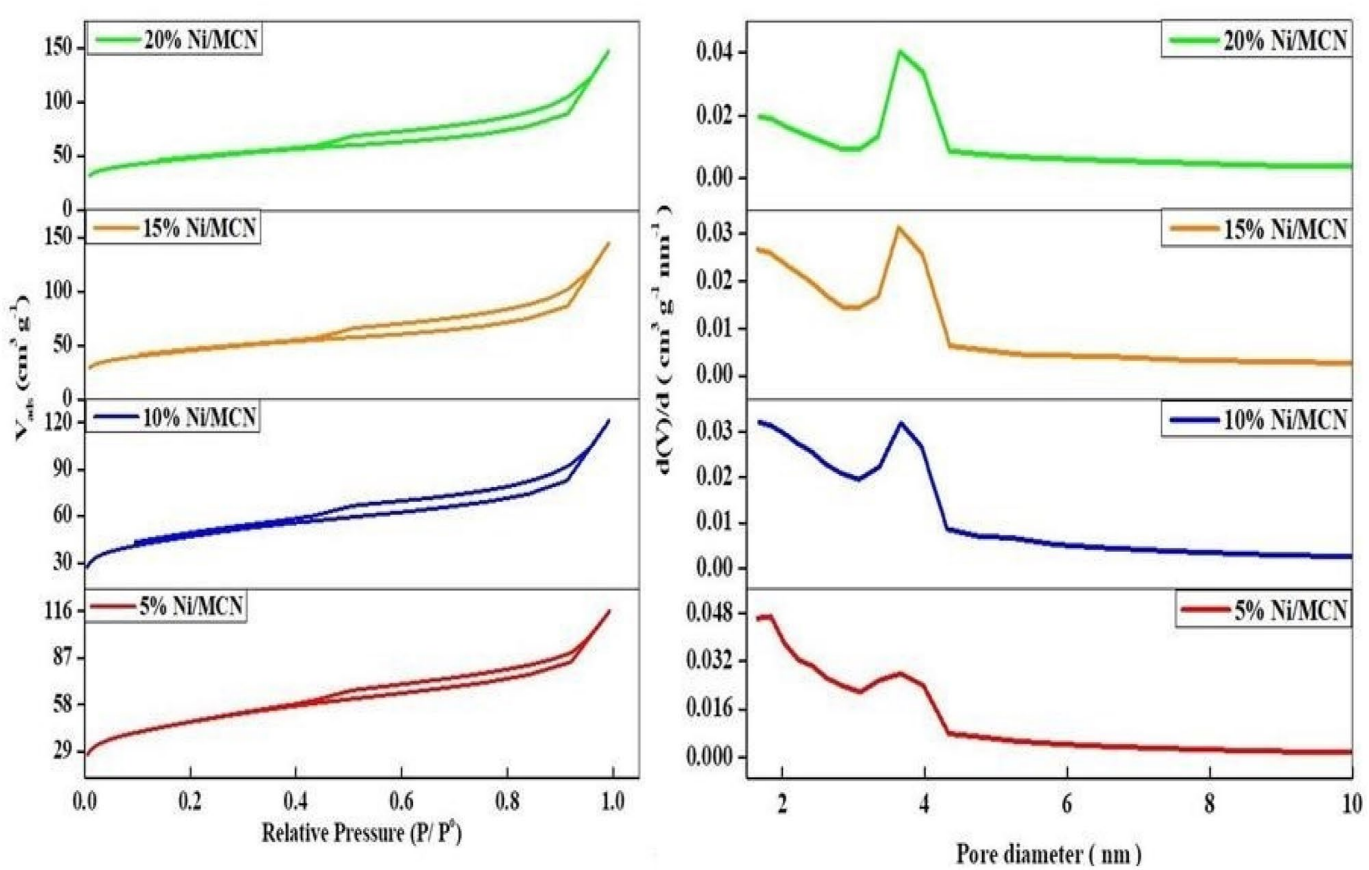
Nitrogen adsorption–desorption isotherm and pore size distribution of the prepared samples.

As shown in the N_2_ adsorption–desorption isotherms, Fig. 2, the parent and the Ni-loaded MCN catalyst have a type IV isotherm with an H_3_ hysteresis loop^59^, which indicates that the pores of the synthesized catalysts were within the mesoporous scale and the capillary condensation occurs at the relative pressure value of 0.45 to 0.9^70^. Furthermore, this type of hysteresis loop produced from materials contains slit-shaped pores as a result of the aggregation of plate-like particles^71^. No significant changes were observed in the shape of the N_2_ adsorption isotherm of the pristine support, affirming the intactness of the original pore structure of the pristine support after Ni loading. Please note that a progressive decrease in the amount of the adsorbed N_2_ is observed with an increase in Ni loading due to the partial occupation of the pores by the Ni NPs.

Table 1 shows the specific surface areas, pore diameters, and pore volumes of the prepared samples. As shown in the table, the pore sizes are within the mesoporous range. As expected, the pure MCN exhibits the largest surface area and total pore volume of 729.47 m^2^ g^−1^, and 1.03 cm^3^ g^−1^, respectively. In contrast, increasing the Ni loading level resulted in a progressive decrease in the BET surface area and pore volume of the Ni-loaded materials since the surface area and pore volume decreased from 729.49 m^2^ g^−1^ and 1.03 cm^3^ g^−1^ in the case of the pristine MCN to 402.89 m^2^ g^−1^ and 0.43 cm^3^ g^−1^ in the case of 20% Ni/MCN, which can be ascribed to the partial occupation of the porous structure of MCN by Ni NPs. It is worth noting that Ni-containing materials still have a relatively high surface area and pore volume, which are decisive factors for high catalytic activity.

**Table 1:** Textural property of the synthesized catalysts.

Materials	Surface area^a^ (m^2^ g^−1^)	Pore diameter^b^ (nm)	Pore volume^c^(cm^3^g^−1^)
MCN	729.47	3.75	1.03
5% Ni/MCN	540.85	3.67	0.53
10% Ni/MCN	490.45	3.66	0.47
15% Ni/MCN	455.22	3.63	0.45
20% Ni/MCN	402.89	3.59	0.43

^a^Determined from BET equation.

^b^BJH desorption average pore diameter.

^c^BJH desorption pore volume.

#### Morphology analysis

**Figure 3:**
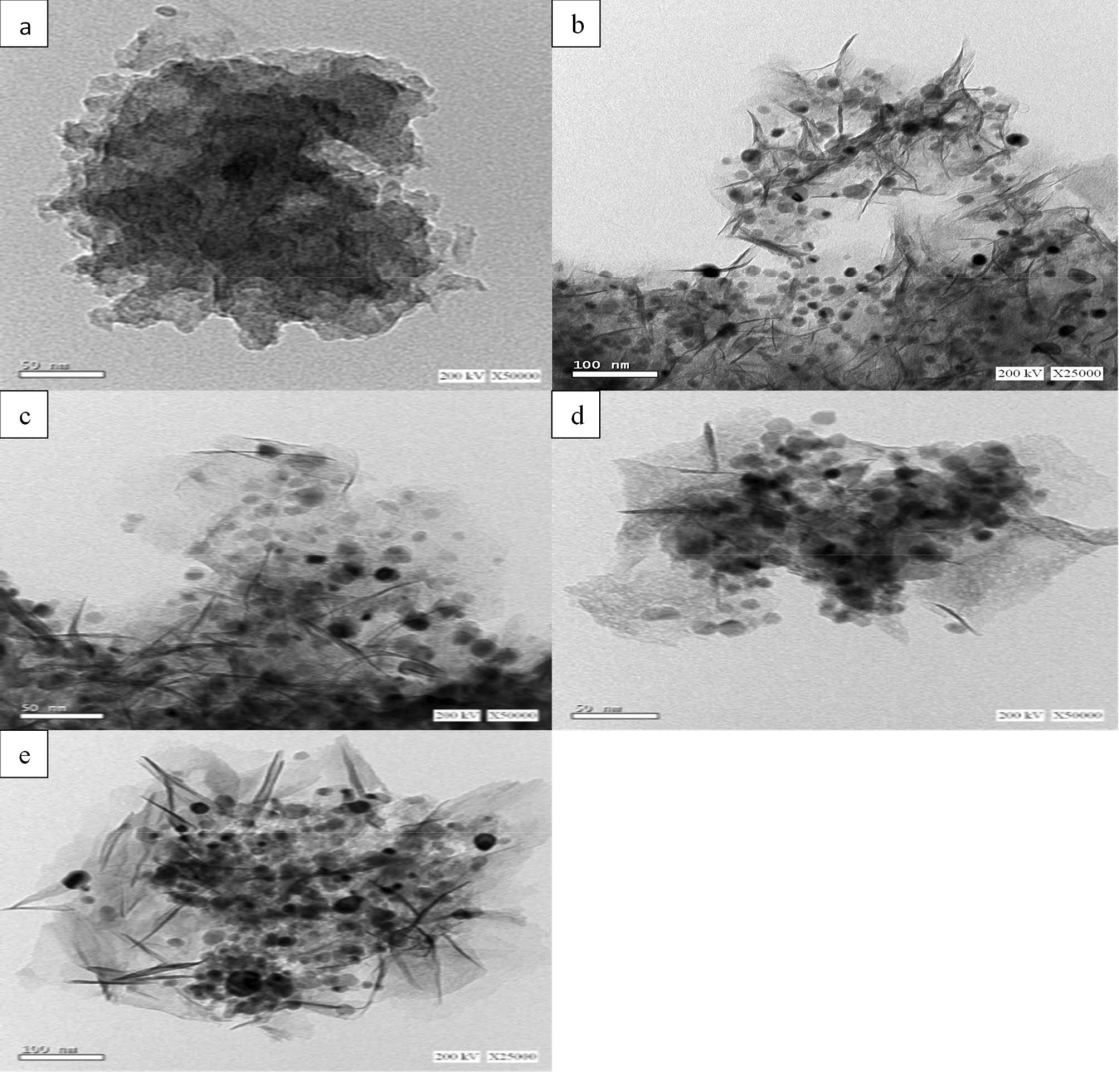
TEM images of (**a**) MCN, (**b**) 5% Ni/MCN, (**c**) 10% Ni/MCN, (**d**) 15% Ni/MCN and (**e**) 20% Ni/MCN.

The TEM technique is employed for examining the morphologies of the catalysts, and the resulting data are shown in Fig. 3. The porous structure of MCN can be confirmed, as indicated by the TEM picture in Fig. 3a. Correspondingly, the nickel-doped catalysts are depicted in Fig. 3b,c,d,e, which demonstrate a thin sheet of the porous structure of MCN with well-dispersed Ni NPs on the surface^59,72^. Considerably, the TEM images of nickel-doped catalysts reveal no evidence of aggregated particles on the surface, confirming the effectiveness of the preparation procedure used.

#### FT-IR spectroscopy

**Figure 4:**
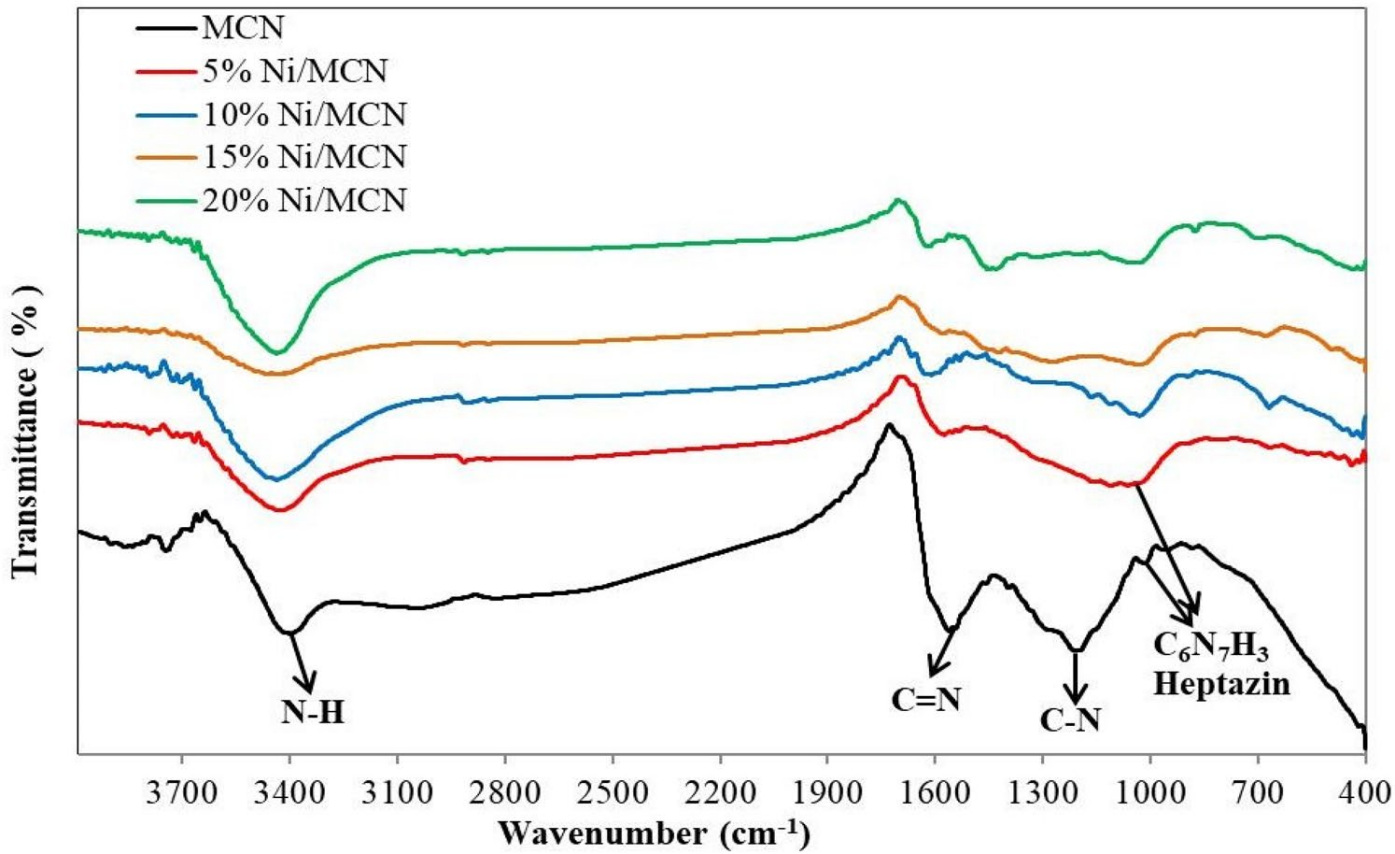
FT-IR of the prepared samples.

The nature of the surface functionalities of the prepared materials was investigated by FT-IR spectroscopy. Both the bare material and the hybrid assembly exhibit typical mpg-C_3_N_4_ features, which can be clearly shown in Fig. 4. The MCN spectrum showed two strong peaks at 1184 and 1535 cm^−1^, which can be assigned to the stretching vibration modes of the aromatic C–N and C=N bonds of the graphitic layers in the MCN skeleton, respectively^64^. In contrast, the spectrum shows that the peak at 1535 cm^−1^ was preserved whereas the peak at 1184 cm^−1^ disappeared upon Ni NPs impregnation. Furthermore, due to the presence of heptazine aromatic rings in the structure, all samples exhibit a breathing mode at around 1000 cm^−1^^65,66^. Furthermore, Both MCN and Ni-doped catalysts displayed a significant peak at 3385 cm^−1^, which was attributed to the N–H or NH_2_ stretching modes in the MCN structure, whereas the retention of this peak in the Ni-doped catalysts indicates excellent impregnation with no defects in the structure of the support^76^. In general, the range at which the N–H stretching mode appears is between 3000 and 3200 cm^−1^ while the sharp peaks with higher wavenumber values as in our case (3385 cm^−1^) suggest a highly ordered structure with few N–H sites as well as limited H-bonding between surrounding units. The peak value may be extended to lower values of wavenumbers as a result of the presence of H-bonds to the N site on adjacent heterocyclic or other interactions. The stretching vibrational modes of some amine groups, such as primary and secondary amines in the sample structure, proved that they formed hydrogen bonds^66,67^, but the shifting of the peak related to N–H to a higher wavenumber value is evidence of the limited H-bond formation in our case. Finally, the addition of nickel alters the peak positions slightly but the spectra of nickel-doped materials are very comparable to those of MCN. These findings prove that nickel was successfully doped on the surface of MCN without creating any structural changes.

#### Raman spectroscopy

**Figure 5:**
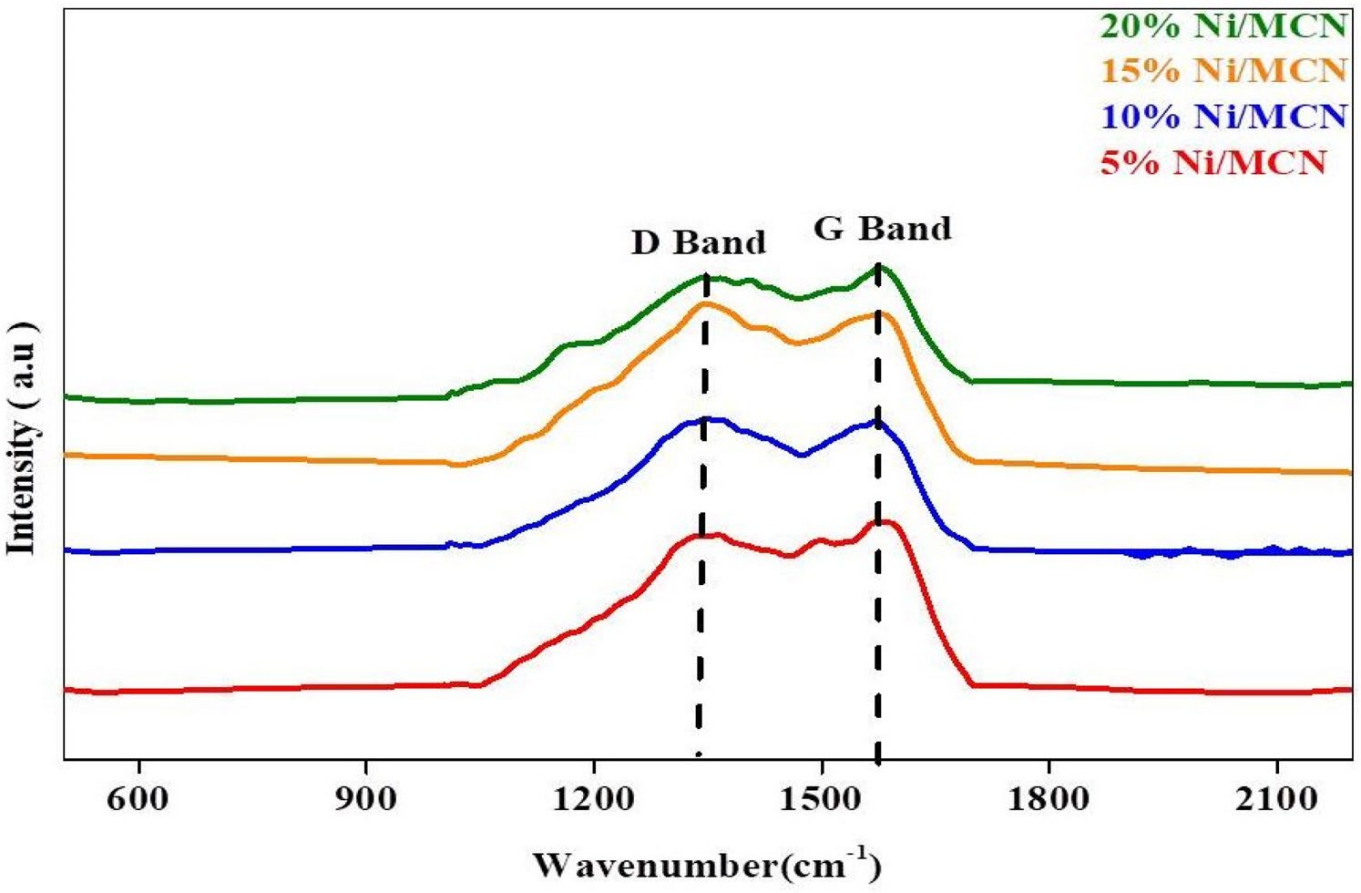
Raman spectra of the prepared samples.

The vibrational properties of the prepared samples were investigated by Raman spectroscopy. MCN's Raman frequencies mode are predicted to be similar to those of analogous unsaturated carbon nitrides, which exhibit about 1500–1600 cm^−1^ for linear chain molecules and 1300–1600 cm^−1^ for closed ring structure molecules^78^. As illustrated in Fig. 5, The Raman frequencies of the Ni-doped mesoporous carbon nitride catalysts exhibited two principal peaks that refer to the D band (disordered) and the G band (graphitized) at 1330 cm^−1^ and 1565 cm^−1^, respectively^79^. It's worth noting that the D band, which becomes active in amorphous materials, refers to the A1g breathing mode, which evolved from the fold aromatic units in the MCN skeleton. In contrast, the G band is due to E2g in-plane stretching vibrations that are associated with the carbon bonds of sp^2^ hybridization in the carbon network of mesoporous carbon nitride^80^.

#### Thermogravimetric analysis

**Figure 6:**
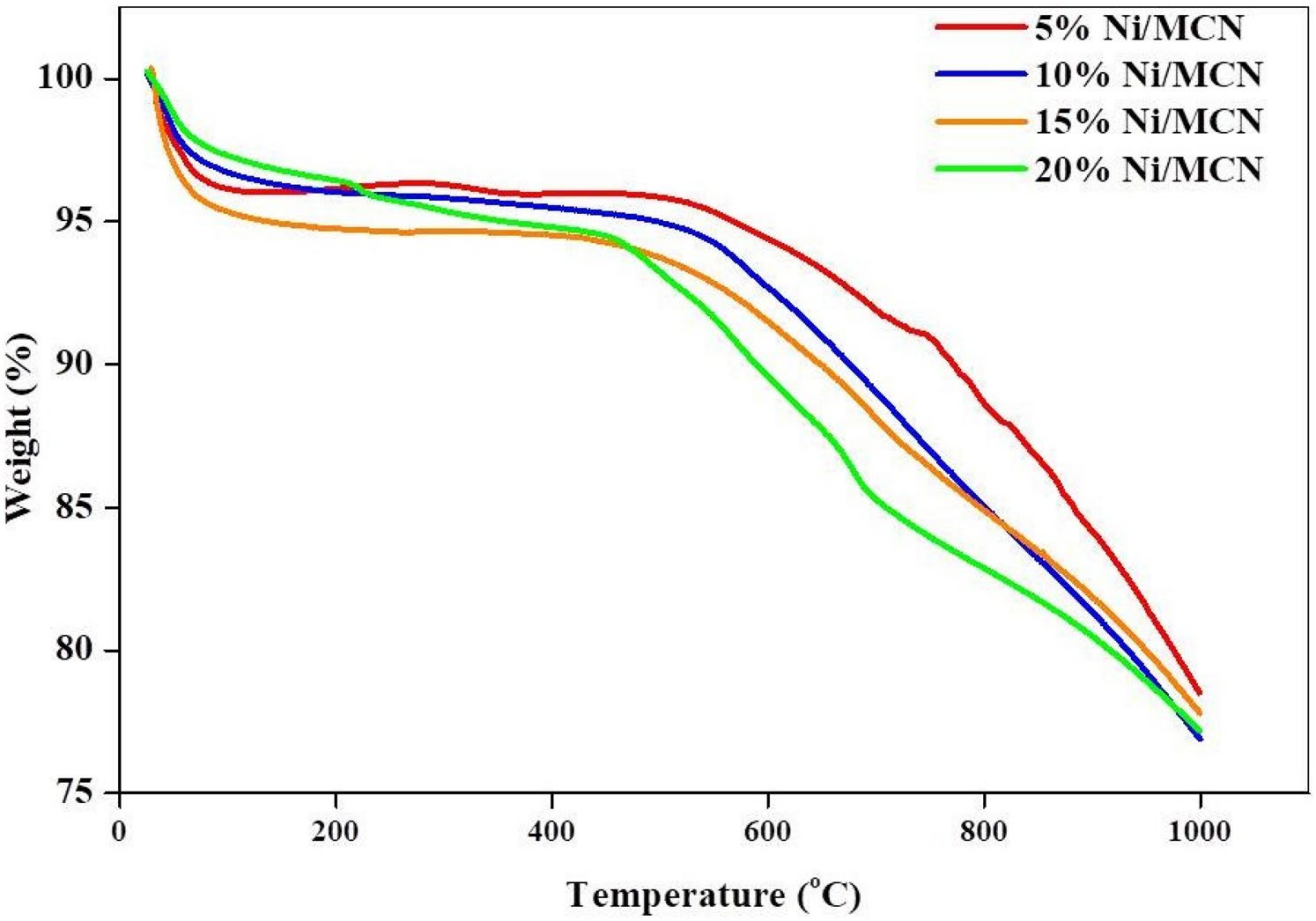
Thermogravimetric (TGA) analysis of the prepared samples.

In terms of implementing the prepared structures in temperature-dependent applications such as CO_2_ methanation, it has been necessary to find out about their thermal stability under operating conditions. The TGA profiles for the catalysts are given in Fig. 6. The TGA analysis of the prepared catalysts showed that samples lost about 4–6% of their weight upon heating from room temperature to 200 °C. This loss is probably due to the removal of adsorbed water, CO_2_, and volatile impurities in the sample structure and pores^81^. Because the reaction temperature range is between 200 and 500 °C and the samples are thermally stable above this range, as shown in Fig. 6, the thermal stability of the catalysts' structure and morphology at temperatures over 500 °C has no effect on our reaction. During heating over the reaction range, the weight of the sample began to drop due to the breakage of the carbon–nitrogen bond^81^. Notably, the samples retain the bulk of their structures at 1000 °C, indicating high thermal stability in general.

#### H_2_ TPR

**Figure 7:**
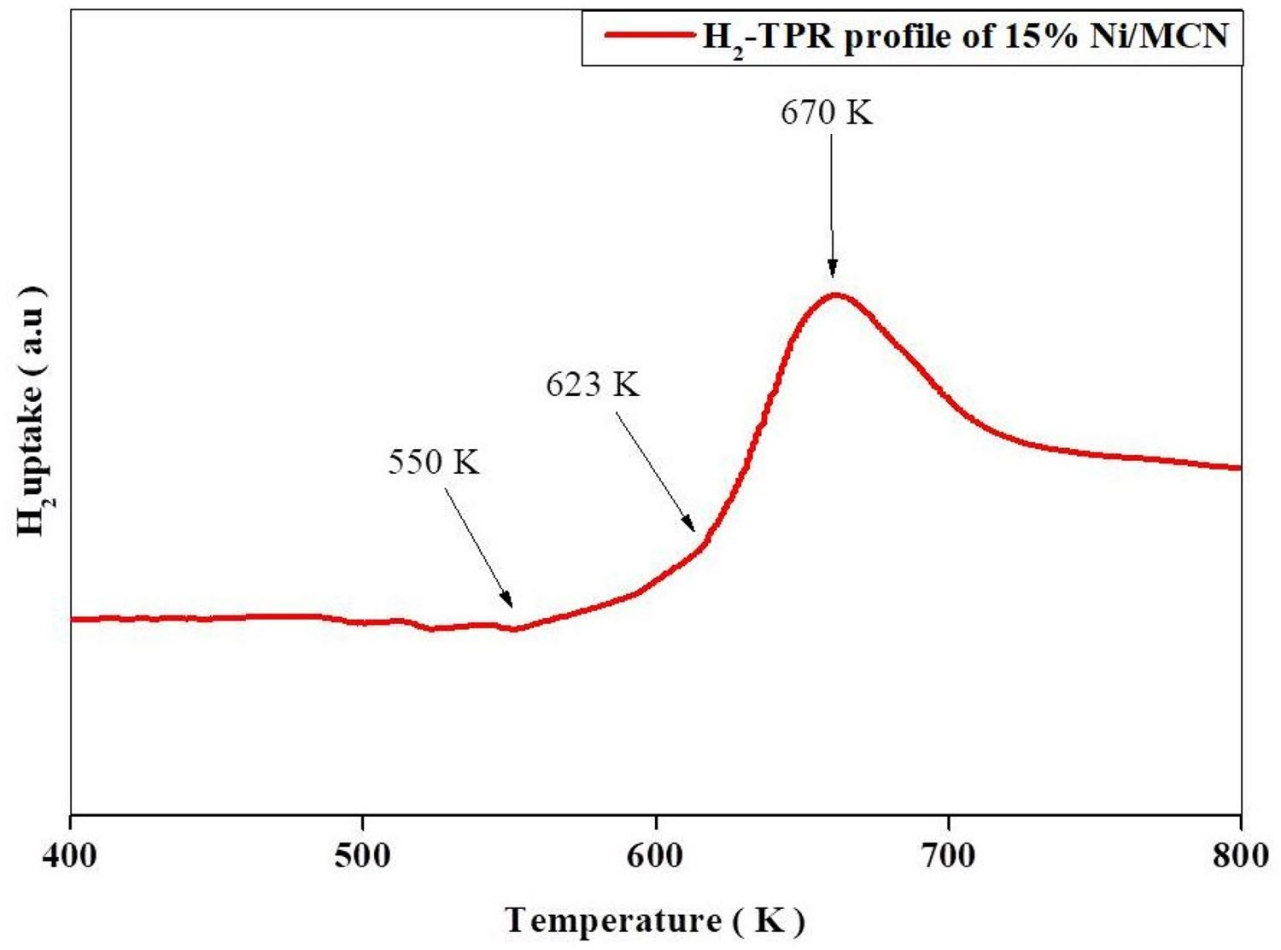
H_2_-TPR profile of 15% Ni/MCN.

To explore the reducibility of a 15% Ni/MCN catalyst and probable Ni particle interactions with MCN, H_2_-TPR was performed on it. First and foremost, as previously demonstrated in the literature, g-C_3_N_4_ support is not reducible in the H_2_ atmosphere. As a result, there is no reduction peak since the structure only comprises carbon and nitrogen. On heat, nevertheless, it begins to vaporize and degrade thermally at around 700 °C^35,43,82^. Figure 7 depicts the H_2_ uptake as a function of temperature for 15% Ni/MCN. A weak peak was detected at about 670 K, which was associated with the presence of NiO impurities^35,81^. It is worth noticing that this NiO-related peak does not exist in the XRD or FT-IR graphs, indicating that it originates from traces of NiO spread over the wide surface area of MCN (455.22 m^2^ g^−1^). Furthermore, the weak uptake above 700 K could be attributed to the beginning of vaporization and decomposition of the wall of mesopores in the support, as previous research has shown that the incorporation of nickel NPs reduces the stability of the nanohybride composition, with decomposition peaks appearing at temperatures ranging from 420 to 590 °C^35,43^. Furthermore, a very weak H_2_ uptake began between 550 and 623 K, which is associated with bulk NiO species that interact sparingly with the support and are referred to as free NiO species. These free species (referred to as α-type species) are easier to reduce at lower temperatures (300–350 °C) than noncrystalline NiO species that extensively interact with the MCN support (referred to as β-type species) (350–500 °C)^58,83^. Additionally, the sample showed an absence of γ-type species reduction peaks, which appear at relatively high temperatures above 500 °C^83,84^.

#### Elemental analysis

A flame atomic absorption spectrophotometer was used to evaluate the nickel loadings on the g-C_3_N_4_ surface. Table 2 clearly shows the homogeneous dispersion of the nickel element, demonstrating the efficient doping of nickel NPs on the surface of MCN.

**Table 2:** Elemental Analysis.

Sample	5% Ni/MCN	10% Ni/MCN	15% Ni/MCN	20% Ni/MCN
Nickel wt%	5.29%	9.64%	14.76%	20.43%

#### CO_2_-TPD analysis

**Figure 8:**
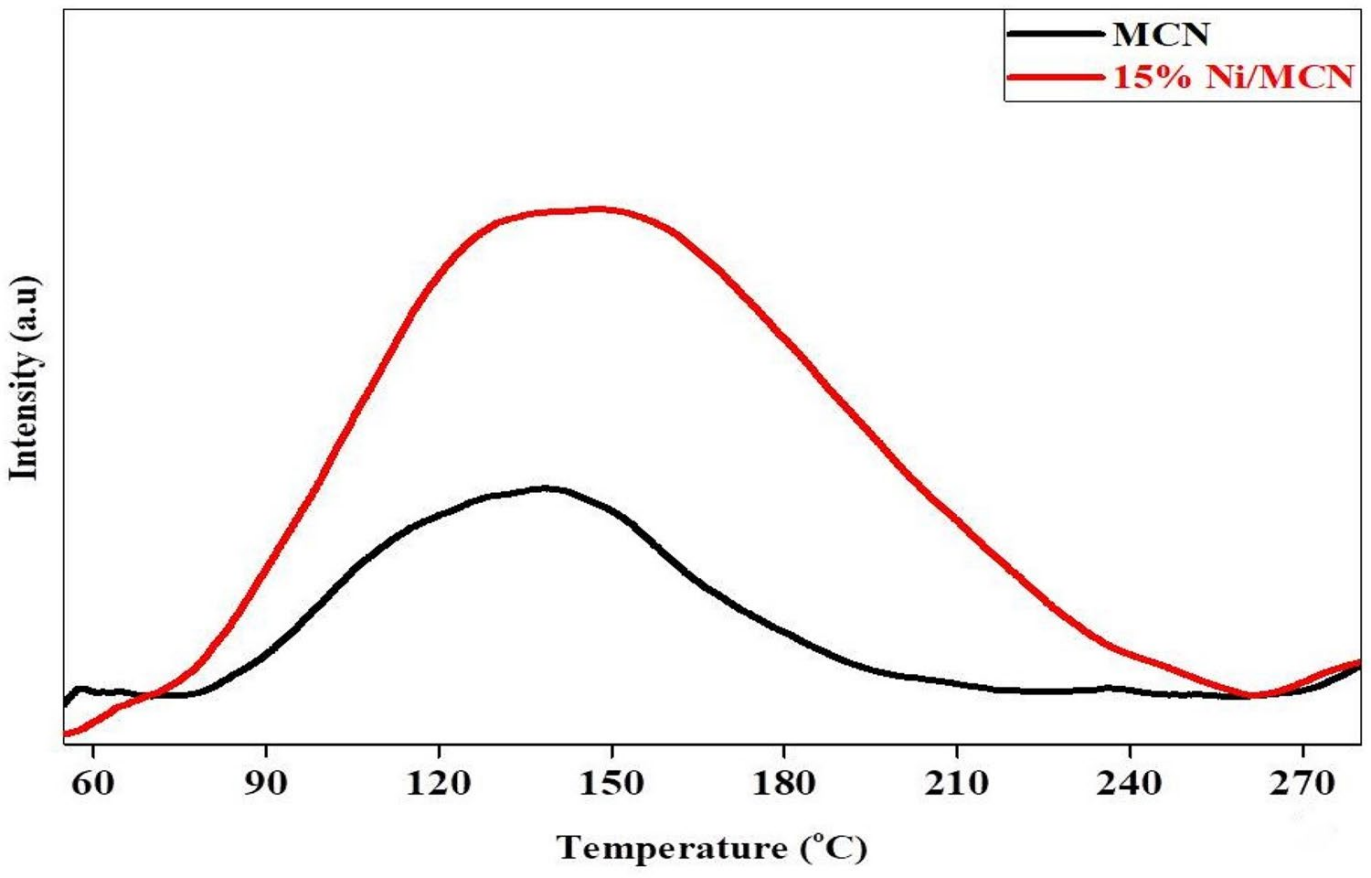
CO_2_-TPD analysis of MCN and 15% Ni/MCN.

In recognition of the inherent integration of nitrogen-containing groups in the primary structure of g-C_3_N_4_, the CO_2_-TPD approach was utilized to assess the basicity and CO_2_ adsorption capability of MCN before and after nickel nanoparticle impregnation. In theory, the MCN structure's nitrogen richness offers ideal electron donors (Lewis base) for CO_2_ molecules, which serve as electron acceptors (Lewis acid). So, a TCD detector is employed in this approach to quantify the desorbed CO_2_ molecules upon heating, which is regarded as explicit evidence of the structural basicity^85^. According to Fig. 8, both MCN and 15% Ni/MCN samples demonstrated an obvious desorption peak in the range 135–150 °C, which was associated with the physisorption and chemisorption of CO_2_ molecules on weak basic sites^86^. Moreover, the nickel-containing sample (15% Ni/MCN) demonstrated a substantially more intense and broad desorption peak as compared with MCN, suggesting that CO_2_ molecules are highly adsorbing on the Ni active sites next to the nitrogen-containing groups in the main structure of MCN. As a result, it is plausible to assume that Ni NPs promote CO_2_ adsorption on the catalyst surface, which improves CO_2_ methanation^87^. Moreover, the desorption peak for 15% Ni/MCN appears at a slightly higher temperature in comparison to MCN, indicating a greater interaction between Ni active sites and CO_2_ molecules as compared to nitrogen sites^86^.

## Catalytic performance

**Figure 9:**
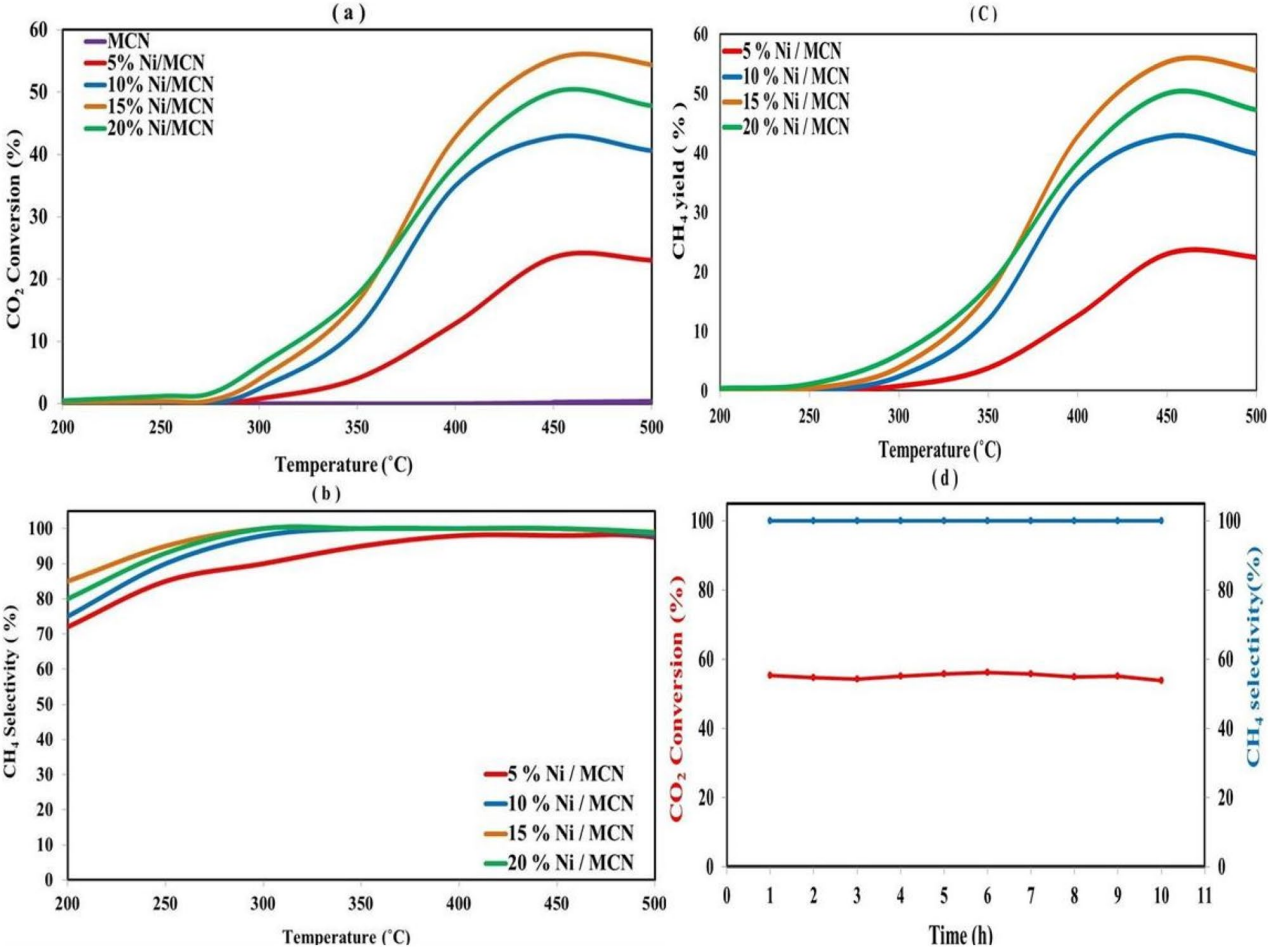
(**a**) CO_2_ conversion capacity of the catalysts at different temperatures, (**b**) CH_4_ selectivity of the catalysts at different temperatures, (**c**) CH_4_ yield of the catalysts at different temperatures and d .short-term stability tests of 15Ni/ MCN at 450 °C and 1 atmosphere.

The catalytic performances of CO_2_ methanation of the prepared catalysts are summarized in Fig. 9, where a, b, c, and d correspond to CO_2_ conversion, CH_4_ selectivity, CH_4_ yield, and short-term stability test, respectively. As shown in Fig. 9 a, CO_2_ Conversion increases by increasing the temperature and then reaches the maximum conversion percent at 450 °C. Subsequently, the conversion begins to decrease at temperatures higher than 450 °C for all samples. The decrease in conversion with a further temperature rising above 450 °C may be attributed to the thermodynamic nature of the CO_2_ methanation process^70,71^. The agglomeration of Ni particles on the MCN surface and carbon–nitrogen bond breaking due to high operating temperature could be additional reasons for the decreased catalytic activity^81,90^. As shown in Fig. 9 a, MCN exhibits very poor catalytic activity, while upon Ni doping, the catalysts showed a noticeably improved conversion. From the results summarized in Table 3, 15% Ni/MCN showed the largest reactivity while the decrease in the case of 20% Ni/ MCN is most probably attributed to the partial pore blocking and surface area decreasing up on nickel increasing on the surface of MCN. On the other hand, the selectivity of methane is shown in Fig. 9b. The catalysts behave like an increase in methane selectivity on temperature rises. The catalysts showed the highest methane selectivity from 350 °C to 450 °C reaches to 100% except 5% Ni/MCN showed relatively lower methane selectivity as compared with 10%, 15%, and 20% Ni/MCN. The selectivity of the prepared catalysts begins to decrease noticeably over 450 °C, which indicates the majority of other reactions, especially the reverse water- gas shift reaction which increases the produced CO gas instead of CO_2_^73,74^. The yield of CH_4_ for all catalysts exhibits the same behavior of the conversion trend. As revealed in Fig. 9c, the methane yield increased by temperature rising where the optimum temperature and maximum yield is at 450 °C. Subsequently, the methane yield begins to decrease upon further heating because of the exothermic character of the reaction^93^. It is worth noting that 15% Ni/MCN provides the largest methane yield while the decrease in the case of 20% Ni/ MCN is a result of pore blocking and accumulation of the Ni NPs on the surface of the MCN. For the short-term stability test, Fig. 9d shows the 10-h stability test of CO_2_ conversion and methane selectivity of the 15% Ni/MCN catalyst as a function of time at optimum temperature (450 °C) and atmospheric pressure^59^. The results show that the CO_2_ conversion stills around the initial result, 55.3%, and the methane selectivity remained around 100% for 10 h. It can be deduced from these experimental results that the catalyst has good stability for CO_2_ conversion and methane selectivity at the operating conditions adopted in this study.

**Table 3:** Catalytic performance of Ni/MCN series at 450 °C.

Materials	MCN	5% Ni/ MCN	10% Ni/ MCN	15% Ni/ MCN	20% Ni/ MCN
CO_2_ Conversion (%)	**0.28**	**23.5**	**42.8**	**55.30**	**50.03**

### Characterization after CO_2_ methanation

**Figure 10:**
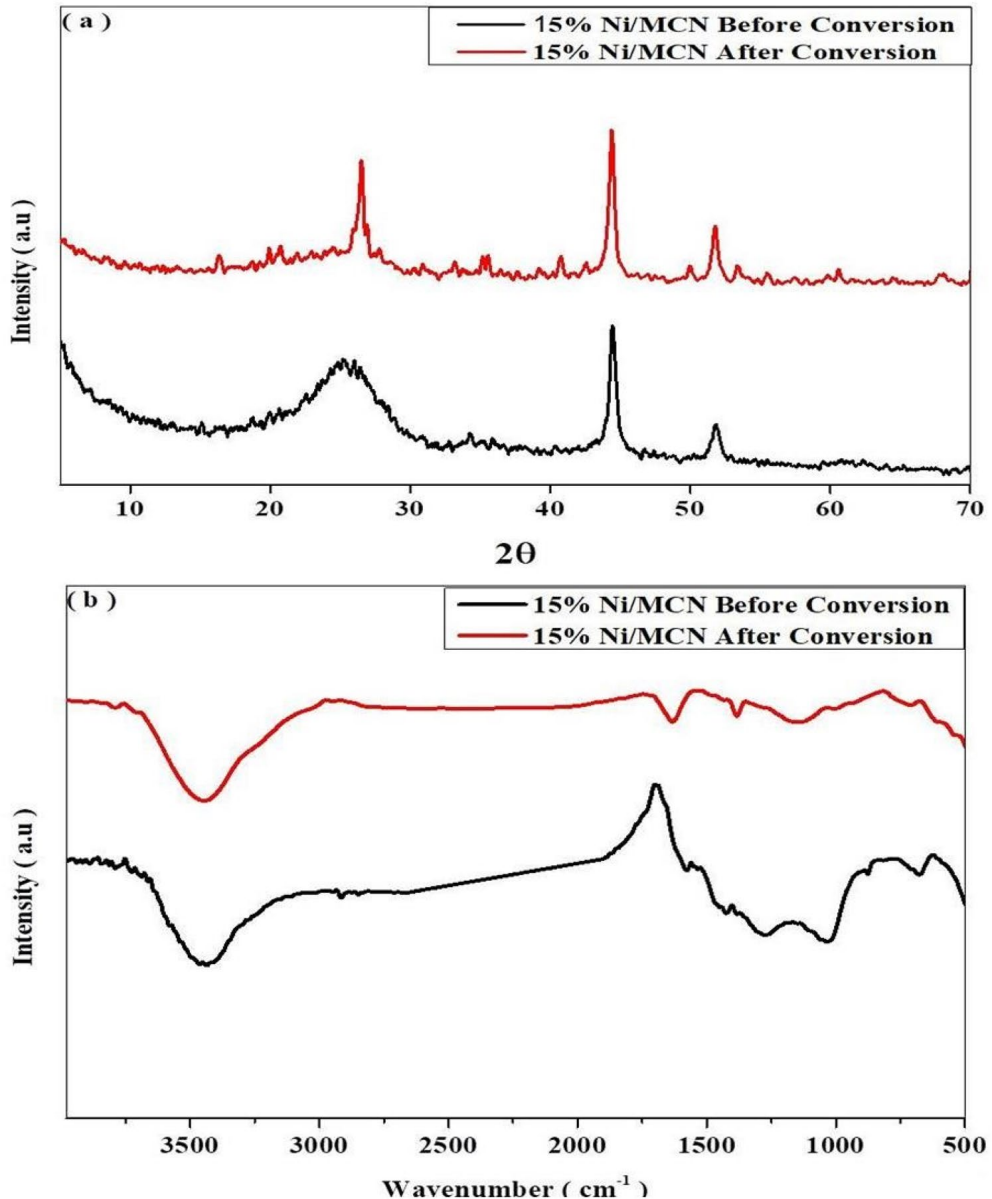
(**a**) XRD of 15% Ni/MCN before and after the CO_2_ methanation, (**b**) FT-IR of 15% Ni/MCN before and after the CO_2_ methanation.

To evaluate the stability of the catalyst after the CO_2_ methanation, XRD and FT-IR were performed on a 15% Ni/MCN catalyst after the stability test. As illustrated in Fig. 10. a the catalyst preserves the peaks associated with nickel nanoparticles at 44.5° and 51.8°, which correspond to the (111) and (200) reflection planes, respectively (PDF no. 01–087-0712, ICDD)^67^. Furthermore, the catalyst preserves the peak at 25° which is related to the MCN with changes in its sharpness and position. The compression of the average interlayer distance of the aromatic systems stacking in the MCN structure is most inevitably responsible for the changes in peak broadening and slightly shifting to 27.3º. These changes may be attributed to the continuous heating at 450 °C for an extended period of time during the stability test, which has an effect on the interlayer distance of the aromatic systems but has no effect on the catalyst's main structure^94^. Figure 10. b then shows that the FT-IR spectra for 15% Ni/MCN before and after CO_2_ methanation are similar. The retention of the peaks at 1000, 1535, and 3385 cm^−1^ for the heptazine aromatic rings, C-N bond stretching, and stretching mode of N–H groups, respectively, indicate the structure's stability following the CO_2_ methanation test.

### Comparison study

In this section, The CO_2_ methanation performance of the 15Ni/MCN catalyst was compared with other Ni-based catalysts that were recently published (Table 4). In the current study, 15Ni/MCN showed the best catalytic activity with 55.30% conversion. As shown in Table 4, this result is consistent with the results published in the literature for the monometallic Ni-based catalysts, which may slightly ascend or descend according to the support nature and reaction conditions difference.

**Table 4:** Monometallic Ni-based catalysts for CO_2_ methanation.

Samples	Preparation method	H_2_/CO_2_	WGHSV (mL g^−1^ h^−1^ )	Pressure (bar)	Reaction temperature (^o^C)	CO_2_ Conversion (%)	^a^Specific activity (mole g^−1^ h^−1^ )	Product distribu-tion	CO_2_ Selectivity ( % )	Ref
10% Ni/MOF-5	Impregnation	4	7500	1	280	47.2	0.28	CH_4_	> 99%	^26^
10% Ni/La_2_O_3_	Impregnation	4	20,000	1	400	53	0.58	CH_4_	97%	^93^
10%Ni/Pr_2_O_3_-CeO_2_	Microwave assisted sol–gel	4	25,000	1	350	54.5	0.80	CH_4_	100%	^95^
10%Ni/MgO-CeO_2_	Microwave assisted sol–gel	4	25,000	1	350	43.2	0.64	CH_4_	100%	^95^
20%Ni/TiO_2_	One-pot sol–gel	4	–	1	400	50	n.a	CH_4_	100%	^96^
10%Ni/CeO_2_-ZrO_2_	AE method	4	20,000	1	275	55	0.59	CH_4_	> 99.6%	^97^
15%Ni/zeolite	Fusion	4	12,000	1	450	53	0.32	CH_4_ CO	> 90%	^98^
15%Ni/ZrO_2_	Combustion	4	48,000	1	300	60	2.21	CH_4_	100%	^99^
15% Ni/ rGO	Incipient wetness impregnation	4	2400	10	240	55.30	0.13	CH_4_	100%	^100^
15%NiLa5	Hydrolysis	4	45,000	1	250	61	2.31	CH_4_	100%	^101^
20%Ni/TiO_2_	sol–gel	4	48,000	1	420	53	1.01	CH_4_	100%	^102^
10%Ni@UiO-66 (DS)	double solvent	8	16,500	1	350	47.5	0.51	CH_4_ CO	83.7%	^103^
10%Ni@MIL-101 (IMP)	Wet impregnation	8	46,500	1	320	56.4	1.80	CH_4_ CO	91.6%	^103^
10%Ni@MIL-101 (DS)	double solvent	8	46,500	1	320	19.2	0.61	CH_4_	63.6%	^103^
20%Ni/SiO_2_	incipient-wetness impregnation	4	–	1	400	54	n.a	CH_4_ CO	92%	^104^
10%Ni-CexZr1-xO_2_	Ammonia evaporation	4	22,000	1	400	55	0. 9	CH_4_	97.5%	^105^
15% Ni/MCN	impregnation	4	120,000	1	450	55.30	4.49	CH_4_	100%	Current work

^a^Specific activity normalized by the weight of the catalyst ( mole of CO_2_/( gram of catalyst* time (h)).

## Conclusion

In this work, a series of Ni nanoparticles supported by mesoporous carbon nitride (5–20 wt. %) catalysts were synthesized successfully by the impregnation method for CO_2_ methanation. The physicochemical properties of the prepared catalysts were characterized by X- ray diffraction, BET, FT-IR, Raman, TEM, TG–DTA analysis, Atomic Absorption spectrophotometer, H_2_-TPR, and, CO_2_-TPd. The proper phase formation and the existence of peaks that related to nickel at higher percentages of doping were investigated by X-ray diffraction while the surface area, pore size distribution, and porosity nature were confirmed by the N_2_ adsorption–desorption isotherm technique. Besides these analysis techniques, the chemical bonds and crystallinity were revealed by FT-IR and Raman spectroscopy respectively. TEM was used to study the morphology of the prepared samples. The thermal stability of the samples was examined by TG–DTA. Additionally, the nickel loading was investigated by atomic absorption spectrophotometer, while the alkalinity of the catalysts was evaluated by the CO_2_-TPD technique. Furthermore, the H_2_-TPR test was used to evaluate the reduction profile of the support and the best catalyst. The catalytic measurements were performed at atmospheric pressure and along a temperature range between 200 – 500 °C. The prepared catalysts showed increasing in CO_2_ conversion, methane yield, and selectivity by raising the reaction temperature from 200 to 450 °C while the activity began to decrease with a further temperature rising to 500 °C. Under kinetically controlled conditions, MCN showed very poor catalytic performance as a result of Ni absence while the CO_2_ methanation improved noticeably up on nickel ratio increasing from 5 to 15% Ni/MCN. CO_2_ conversion was 0.28, 23.5, 42.8, and 55.30% for MCN, 5% Ni/MCN, 10% Ni/MCN, and 15% Ni/MCN respectively. The catalytic conversion showed a slight decrease by increasing the Ni loading whereas 20% Ni/MCN showed 50.03% CO_2_ Conversion at the optimum condition. This decrease in catalytic performance may probably relate to the pore blocking by Ni nanoparticles. The prepared catalysts exhibit high methane selectivity and good methane yield at the optimum reaction temperature (450 ºC) reached to almost 100% and 55.30% for selectivity and yield respectively for the best catalyst (15% Ni/MCN). In the short-term stability study, 15% Ni/MCN showed good and stable results for CO_2_ methanation and methane selectivity.

### Ethical approval

This article does not contain any studies with human participants or animals performed by any of the authors.

## Data Availability

The datasets generated and/or analysed during the current study are available in the Chemistry and Chemical biology repository, Crystallography Open Database (COD). COD ID is 1,534,042 (mesoporous carbon nitride C_3_N_4_). COD ID is 1,534,892 (Nickel nanoparticles). Link: http://www.crystallography.net/cod/.
